# Who is research serving? A systematic realist review of circumpolar environment-related Indigenous health literature

**DOI:** 10.1371/journal.pone.0196090

**Published:** 2018-05-24

**Authors:** Jen Jones, Ashlee Cunsolo, Sherilee L. Harper

**Affiliations:** 1 Department of Geography, University of Guelph, Guelph, Ontario, Canada; 2 Labrador Institute, Memorial University, Happy Valley-Goose Bay, Newfoundland Labrador, Canada; 3 Department of Population Medicine, University of Guelph, Guelph, Ontario, Canada; University of Western Australia, AUSTRALIA

## Abstract

**Background:**

Addressing factors leading to health disparities in the Circumpolar North require approaches that consider and address the social determinants of health including on-going colonization. Today, colonization and related policies and processes, continue to manifest in the marginalization of Indigenous knowledge, particularly its use in research; however, Indigenous populations have moved from being research subjects to leaders and consumers of environmental health research. Given the tensions that exist between how health research is conducted, how the results are mobilized, and who has control and access to the results, we examine how peer-reviewed environment-related Indigenous health research in the Circumpolar North is serving the needs of Indigenous communities, governments, and organizations.

**Methods:**

A modified systematic-realist literature review was conducted. Three databases were searched for peer-reviewed literature published from 2000 to 2015. Articles were included if the research focused on the intersection of the environment and health in Northern Canada and/or Alaska. A total of 960 unique records were screened for relevance, and 210 articles were analysed.

**Results:**

Of these relevant articles, 19% discussed how Indigenous peoples were engaged in the research. There was a significant increase in reporting participatory, community-based methods over time; the proportion of articles reporting community-engagement varied by research topic; quantitative research articles were significantly less likely to report community-engaged methods; and most articles did not clearly report how the results were shared with the community.

**Conclusion:**

The results raise a number of questions for the field of Circumpolar environment-related Indigenous health research, including whether or how authors of peer-reviewed literature should (or should not) be obliged to describe how research is serving Northern Indigenous communities. The results are intended to stimulate further conversations and bridge perceived dichotomies of quantitative/qualitative, Western/Indigenous, and empirical/community driven research approaches, as well as underlying assumptions that frame health research.

## Introduction

Despite the heterogeneity of Indigenous cultures and communities across the Circumpolar North, there is often a shared experience of health inequities stemming from on-going colonization, including environmental displacement and dispossession, forced relocation, language and cultural erosion, and intergenerational trauma [1–12]. Inequities resulting from these socio-political realities include higher rates of infant mortality; infectious and chronic diseases; suicide; and lower life expectancies [6, 13, 14]. A challenge to those working in the health field—including policy makers, practitioners, community leaders, and frontline workers—is to understand and promote health within these socio-political contexts [9–11, 15].

Legacies of colonization manifest in the systemic marginalization of Indigenous knowledge, particularly its use in research [14, 16, 17]. For example, researchers have posited that the epistemological and praxiological divides between Indigenous knowledge and empirical positivist thought have manifested in research approaches [18–22]. In many cases, empirical approaches to health research are challenged to consider a holistic understanding of Indigenous health [19]. Indeed, metrics applied to measure Indigenous health often do not reflect Indigenous conceptualizations of health, as these metrics often originate in Western-informed reductionist research approaches. Such health research approaches may reify the objectification and marginalization of Indigenous knowledge contributions through the positioning of Indigenous knowledge as anecdotal, tokenistic, or unscientific, supporting the erasure of Indigenous knowledge from consideration [23–25]. Indeed, many Indigenous communities have expressed experiences of being ‘researched to death,’ positioned only as subjects of research, resulting in research that does not respond to local priorities, nor incorporate Indigenous ways of knowing [16, 21, 26–28].

Indigenous peoples worldwide, however, have not sat idly watching research define Indigenous health disparities; Indigenous groups and researchers are challenging Western-informed approaches to research [21, 24, 29]. Today, many Indigenous organizations are positioned to develop research projects through meaningful partnerships with researchers, universities, and/or institutes. Indeed, modern-day treaties have positioned Indigenous communities and governments to increasingly take control over the direction of Indigenous health research [21, 30, 31]. Still, tensions exist between how health research is conducted, how the results are mobilized, and who has control and access to the research results [17, 26, 32]. Additionally, research is often fraught with challenges that may act as barriers for Indigenous community-led health research, including: lack of funding to support community engagement in proposal development; timelines that do not allow for community engagement in research decision making processes; the frequent requirement that the principle investigator must be affiliated with a university; and tensions between qualitative and quantitative approaches [20, 29, 33, 34].

This literature review uses a systematic realist approach (defined below) to consider these challenges, and aims to understand how peer-reviewed environment-related Indigenous health research conducted in Northern Canada and the United States (Alaska) is serving the needs of Indigenous communities, governments and organizations. Specifically, we ask the following questions of literature focused on Northern environment-related Indigenous health:

1. Does Northern environment-related Indigenous health research speak to priorities communicated by Indigenous communities, governments, and organizations? How is this communicated in articles?
2. How can Indigenous communities, governments, and organizations access articles about environment-related health research conducted in the communities that they serve?
3. How do authors communicate the way in which the research results were used or applied by Indigenous communities, governments, or organizations?

We seek to stimulate conversations between and among health researchers, Circumpolar Indigenous communities, governments, health practitioners, and policy makers to identify methodologies that draw upon strengths from different research approaches; promote engagement with the communities, organizations, and governments; and respond to health and environment priorities of a changing North. While we examined research from the Circumpolar North, the findings, implications, and calls for future discussion apply to other Indigenous communities worldwide.

## Materials and methods

This research was conducted through a systematic realist literature review approach (SRLR) [35–39]. Systematic literature reviews identify, screen, and synthesize large amounts of data through a transparent, comprehensive, and reproducible process [38]. A systematic realist literature review focuses on analyses of the underlying context of the literature, synthesis of data, and analysis of data by asking the *who*, *what*, *why*, and *how* questions of the literature [36, 40]. A realist review method considers the larger context of the concepts, constructs, motivations, and underlying theory that are shaping the research articles [36]. As such, a systematic realist literature review facilitates an analysis that is more explanatory in nature, discovers crosscutting themes across the literature to deepen understanding, strengthens critical analysis of the topics studied, and searches for common underlying mechanisms [35–37].

This SRLR drew upon methods outlined in the Preferred Reporting Items for Systematic reviews and Meta-Analyses (PRISMA), and drawing from concepts outlined in the PRISMA-Equity reporting guidelines to systematically search the literature and identify relevant articles, while the realist approach was used to analyze and synthesize the articles included for review [37, 38, 41, 42]. The realist approach was operationalised in this review by evaluating each article against community engagement criteria (see below) as a proxy for whom research was serving.

### Search strategy

**Fig 1:**
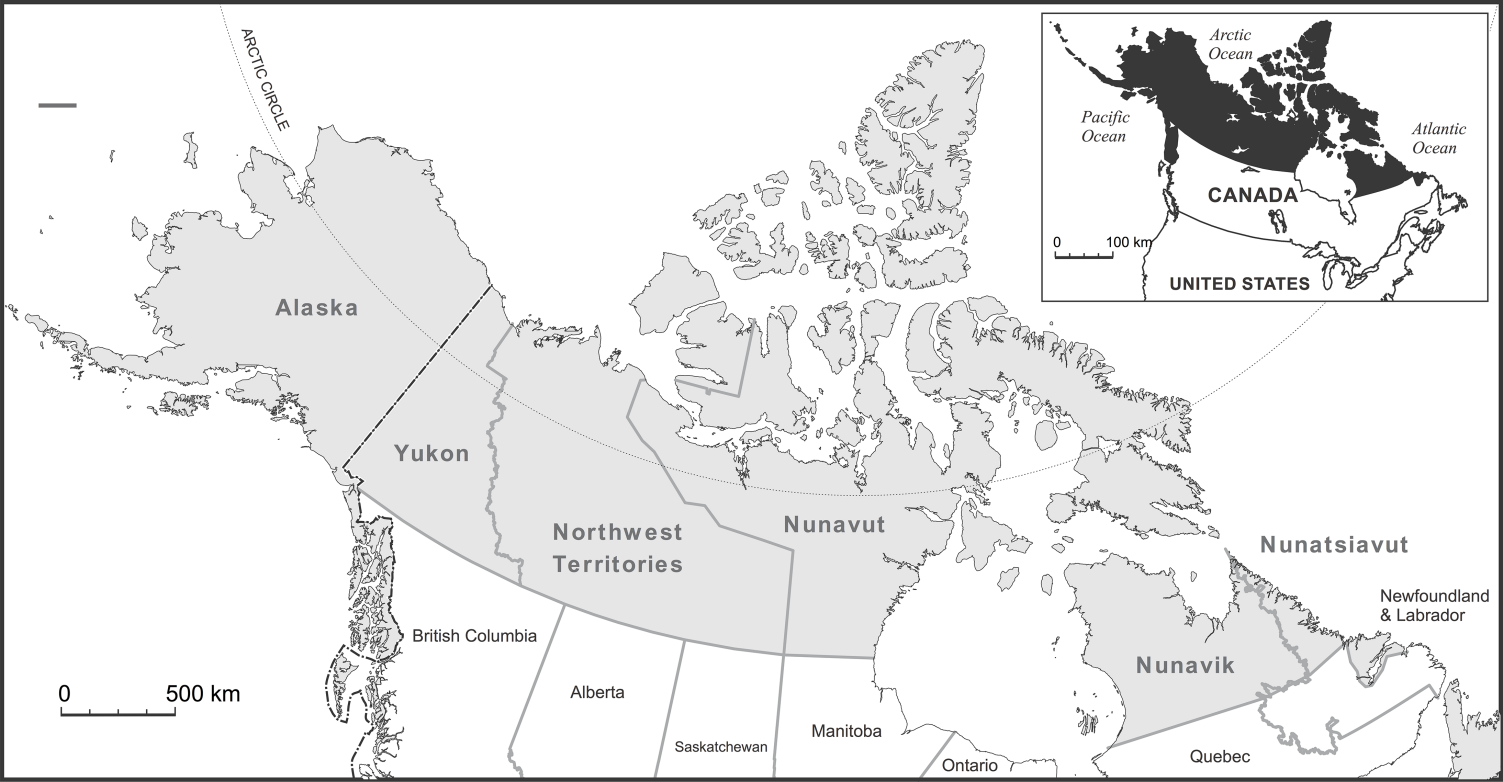
Geographical focus of the systematic realist literature review: Northern Canada and Alaska, USA.

A search strategy aimed to identify English-language peer-reviewed articles published between January 1, 2000 and August 30, 2015 about research conducted in Northern Canada and Alaska (Fig 1), focusing on environment-related Indigenous health issues. In consultation with a health research librarian, a search string was developed (Table 1) and used to search PubMed, Web of Science, and CAB Direct. Records were uploaded into EndNote X7®, and de-duplicated.

**Table 1:** The search terms used in three electronic bibliographic databases to identify articles for the systematic realist literature review on circumpolar environment-related Indigenous health research (2000–2015).

**Bibliographic Databases**	**Search String**
**PubMed**	((environment*) AND ((Circumpolar OR arctic OR Alaska OR “Northern Canada” Or Nunavut OR “Northwest Territories” OR Yukon OR Nunatsiavut OR Nunavik OR “Inuit Nunangat” OR Inuvialuit))) AND ((((Aboriginal OR indigenous OR “first nation*” OR Inuit* OR Inupiat* OR Eskimo* OR Aleut OR native OR Kalaallit OR Tlingit OR tsimshian OR haida OR Gwitchin OR Dene OR athabaskan* OR esquimaux))) OR (("Indians, North American"[Mesh]) OR "Inuits"[Mesh])) Filters: Publication date from 2000/01/01 to 2015/12/31; English
**CAB Direct**	((Circumpolar OR arctic OR Alaska OR “Northern Canada” Or Nunavut OR “Northwest Territories” OR Yukon OR Nunatsiavut OR Nunavik OR “Inuit Nunangat” OR Inuvialuit) AND (Aboriginal OR indigenous OR “first nation*” OR Inuit* OR Inupiat* OR Eskimo* OR Aleut OR native OR Kalaallit OR Tlingit OR tsimshian OR haida OR Gwitchin OR Dene OR athabaskan* OR esquimaux) AND (health) AND la:(En OR English) AND yr:[2000 TO 2015]) NOT ((animal))
**Web of Science**	(Circumpolar OR arctic OR Alaska OR “Northern Canada” Or Nunavut OR “Northwest Territories” OR Yukon OR Nunatsiavut OR Nunavik OR “Inuit Nunangat” OR Inuvialuit) *AND* TOPIC: (Aboriginal OR indigenous OR “first nation*” OR Inuit* OR Inupiat* OR Eskimo* OR Aleut OR native OR Kalaallit OR Tlingit OR tsimshian OR haida OR Gwitchin OR Dene OR athabaskan* OR esquimaux) *AND* TOPIC: (health) *AND* TOPIC: (environment) Timespan: 2000–2015. Search language = English

### Relevance screening and eligibility criteria

Relevance screening consisted of two steps. First, titles, abstracts, and keywords were screened for relevance. Relevant records proceeded to a second step, which involved screening the full-text of each article. In both steps, records were reviewed by two independent reviewers against inclusion/exclusion criteria that were developed *a priori* (Table 2). Reviewers met to discuss and resolve conflicts. All relevant full-text articles proceeded to data extraction and analysis.

**Table 2:** Summary of inclusion and exclusion criteria used to identify circumpolar environment-related Indigenous health literature (2000–2015).

CRITERIA		LEVEL 1 SCREENING: Screening Titles and Abstracts	LEVEL 2 SCREENING: Screening the Full Text of Articles
**Geographical Criteria**	• The study occurred only in Nunatsiavut, Nunavik, Nunavut, Inuvialuit, and/or Alaska (see Fig 1).	✓	✓
	• The study did not include a non-circumpolar region (e.g. studies examining Canada would be excluded, even if Nunavut was covered in the study).	✓	✓
	• The study location was unclear.	✓	
**Human Health Criteria**	• Any facet of human health and/or wellbeing was mentioned.	✓	✓
	• A focus on human health was unclear.	✓	
**Population Criteria**	• The study included Indigenous peoples.	✓	✓
	• The study did not include non-Indigenous peoples.	✓	✓
	• The study population was unclear.	✓	
**Language and Date Criteria**	• An English version of the title and/or abstract was available.	✓	✓
	• The study was published during or after 2000.	✓	✓
**Study Design Criteria**	• The article was a primary research study (e.g. a literature review, commentary, and non-research articles are excluded).		✓
	• The article was peer-reviewed.		✓

### Data extraction & analysis

Data were extracted from relevant articles (S1 Fig, S1 Table). Exact logistic regressions examined the effect of variables (i.e. year, location, research topic, methodologies) on the odds of articles reporting community-engaged approaches (i.e. Indigenous authorship, acknowledging Indigenous contributors, explicitly stating the use of participatory methods, obtaining permission by and participation from local organizations, and results-sharing frequencies and strategies).

Articles that described how Indigenous communities, governments, and/or organizations were engaged in the research process were thematically analysed. In the qualitative thematic analysis of the articles that described a community’s engagement with the research, indicators were developed based on the “4Rs” of Indigenous research—respect, relevance, reciprocity and responsibility—and the First Nations-developed research principles of ownership, control, access and possession (OCAP) [16]. Codes captured: community partners or organizations engagement in the research; initiation of research and by whom; limitations identified by the author(s) related to working with a community or region; research use by the community; and description of results mobilization within the community or with the partnering Indigenous organization. A “risk of bias” or other quality assessments were not conducted on individual studies because we were not assessing treatment effect or effect sizes; rather, we aimed to examine how articles reported environment and health literature serving Indigenous governments and organizations.

## Results

### Description of circumpolar environment-related Indigenous health literature

**Fig 2:**
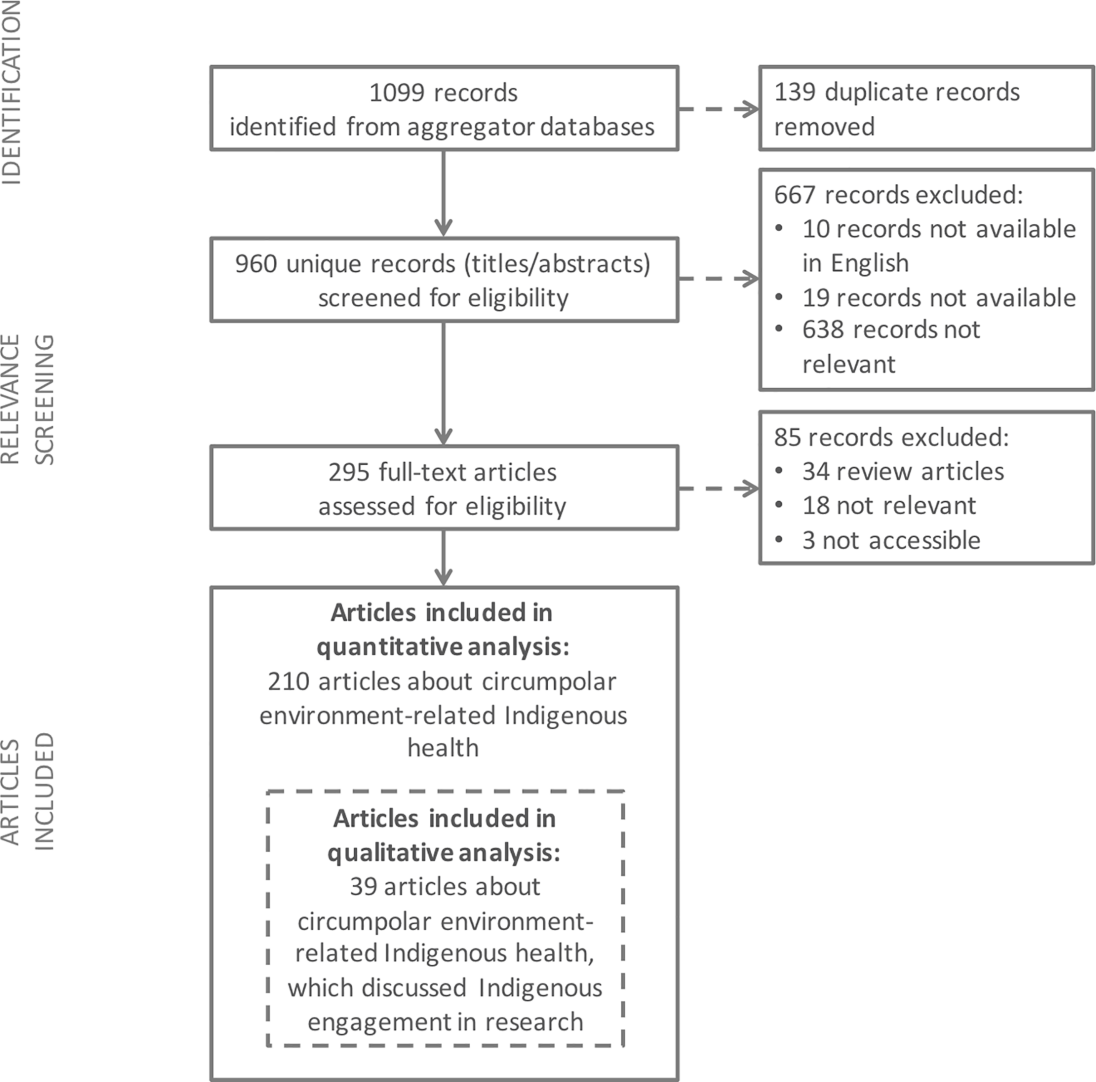
Search strategy and results for the systematic realist literature review. The inter-rater reliability for the screening was κ = 0.87, indicating “strong” agreement among the independent reviewers. [43].

A total of 210 articles met the inclusion criteria (Fig 2). From these environment-related Indigenous health research articles (n = 210); the number of publications increased over time; most studies took place in Nunavut (28.1%), Nunavik (27.6%), and/or Alaska (26.7%); the most common topic was environmental contaminants (41.9%) and the majority of articles used quantitative research methods (75.2%) (Table 3). Community-engaged research was mentioned in 80 articles (38%).

**Table 3:** A summary of article attributes for all studies that examined health and environments in the Circumpolar North (2000–2015).

Article Attributes	Total number of articles	Proportion of all health and environment articles (%)	Number of Articles Reporting Participatory Methods	Proportion of Articles Reporting Participatory Methods in the Category (%)
**Participatory, community-based, or other methods that engage the community in the study were used**	80	38.10%		
Region*				
Nunavik	58	27.60%	9	15.52%
Nunavut	59	28.10%	35	59.32%
Northwest Territories	40	19.00%	25	62.50%
Nunatsiavut	30	14.30%	22	73.33%
Yukon	17	8.10%	10	58.82%
Alaska	56	26.70%	17	30.36%
**Year of Publication**				
2000	3	1.40%	0	0.00%
2001	5	2.40%	0	0.00%
2002	4	1.90%	2	50.00%
2003	5	2.40%	0	0.00%
2004	7	3.30%	3	42.86%
2005	8	3.80%	2	25.00%
2006	17	8.10%	5	29.41%
2007	9	4.30%	6	66.67%
2008	14	6.70%	3	21.43%
2009	12	5.70%	4	33.33%
2010	19	9.00%	10	52.63%
2011	24	11.40%	11	45.83%
2012	22	10.50%	10	45.45%
2013	24	11.40%	7	29.17%
2014	25	11.90%	12	48.00%
2015	12	5.70%	5	41.67%
Topic*				
Environmental contaminants	88	41.90%	18	20.45%
Nutrition and food security	71	33.80%	47	66.20%
Environmental risks and resilience	39	18.60%	30	76.92%
Climate change impacts on health	22	10.50%	20	90.91%
Environment-related unintentional injury	14	6.70%	11	78.57%
**Methods**				
Quantitative	158	75.20%		
Qualitative	38	18.10%	30	78.95%
Qualitative and quantitative methods	14	6.70%	40	25.32%
**Open-access articles**	100	47.60%	50	50.00%
**Indigenous organization(s) was/were listed as a co-author**	60	28.60%	36	60.00%
**Community members or governments were mentioned in the acknowledgment section**	142	67.60%	68	47.89%
**Permission from a local organization was explicitly reported**	106	50.50%	66	62.26%
**Article reported how the results were shared with the community**	31	14.80%	29	93.55%

*These categories are not mutually exclusive and therefore add up to more than 100%.

A total of 80 articles were screened for thematic analysis, after exclusions, 39 articles (18.6%) were reviewed for detailed descriptions of how Indigenous communities, governments, or organizations were engaged in the research (Fig 2).

### Does published research speak to Northern priorities?

#### Articles reporting community-engagement varied by time, location, topic, and method

**Fig 3:**
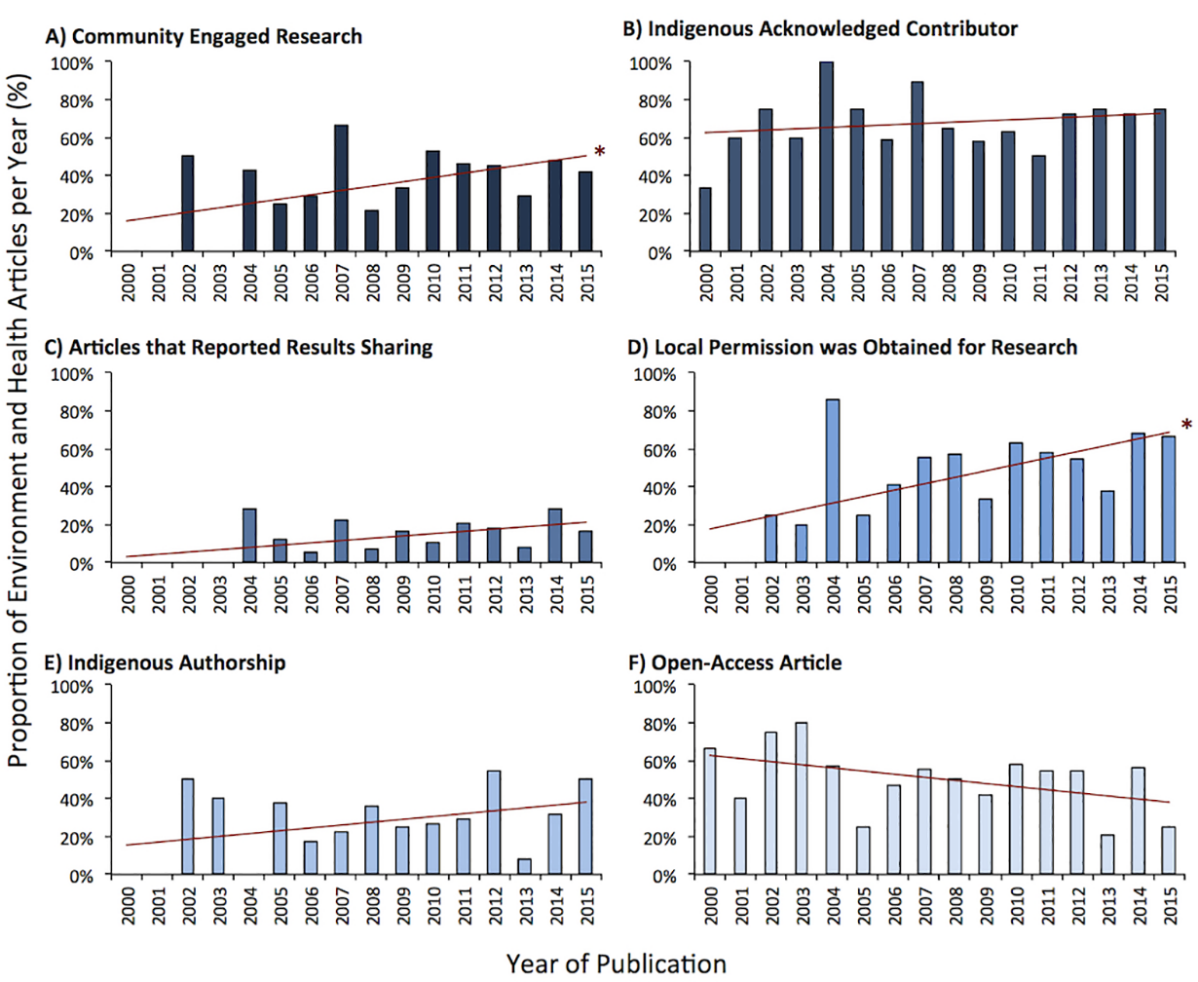
Circumpolar health and environment article attributes that indicate some form of community involvement over time with a linear trend line. Significant trends over time are marked with “*”.

**Fig 4:**
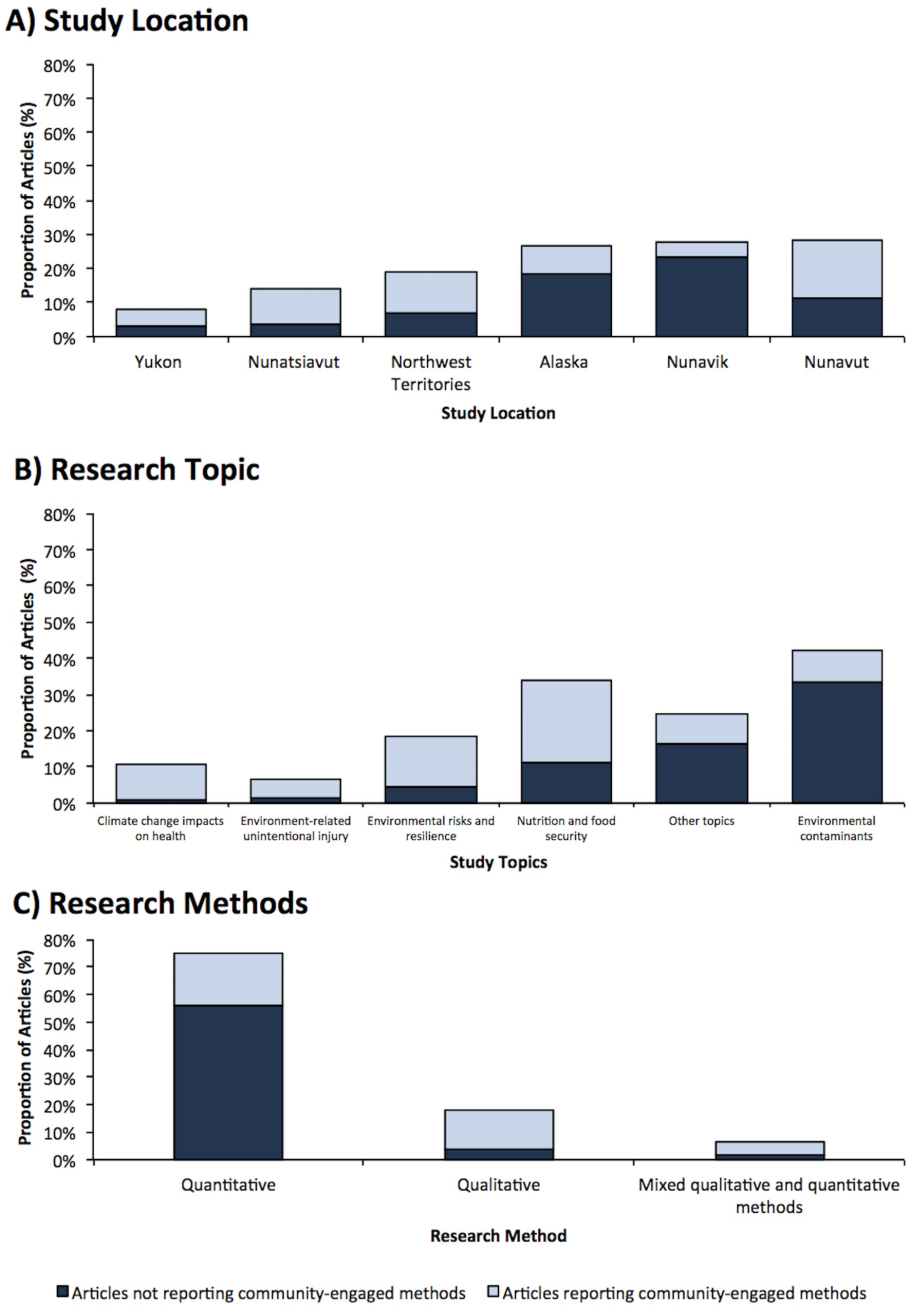
Proportion of circumpolar health and environment articles reporting community-engaged methods by (A) region, (B) research topic, and (C) research methods.

**Fig 5:**
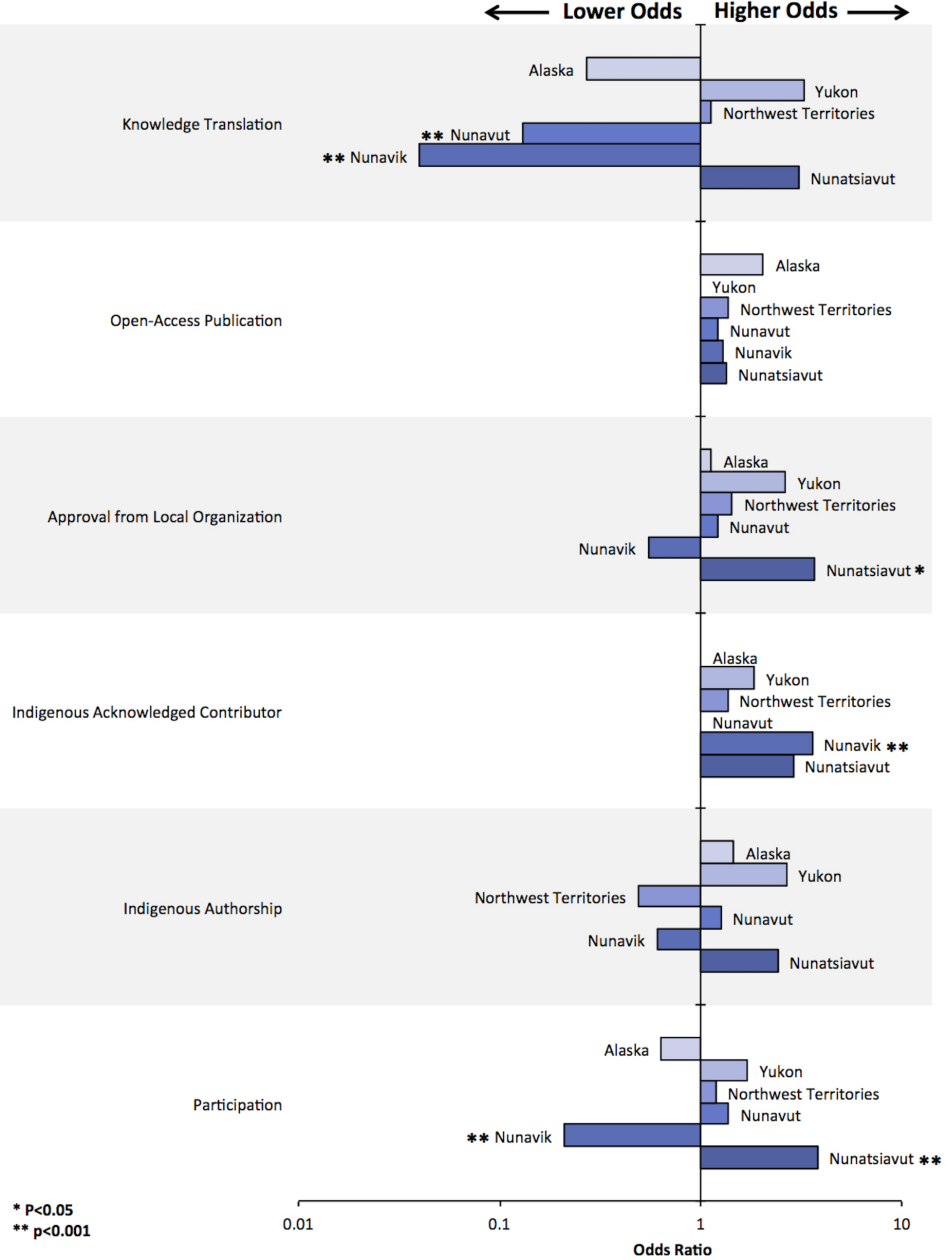
Multivariable exact logistic regression examining associations between location and various indicators of community-engaged research.

**Fig 6:**
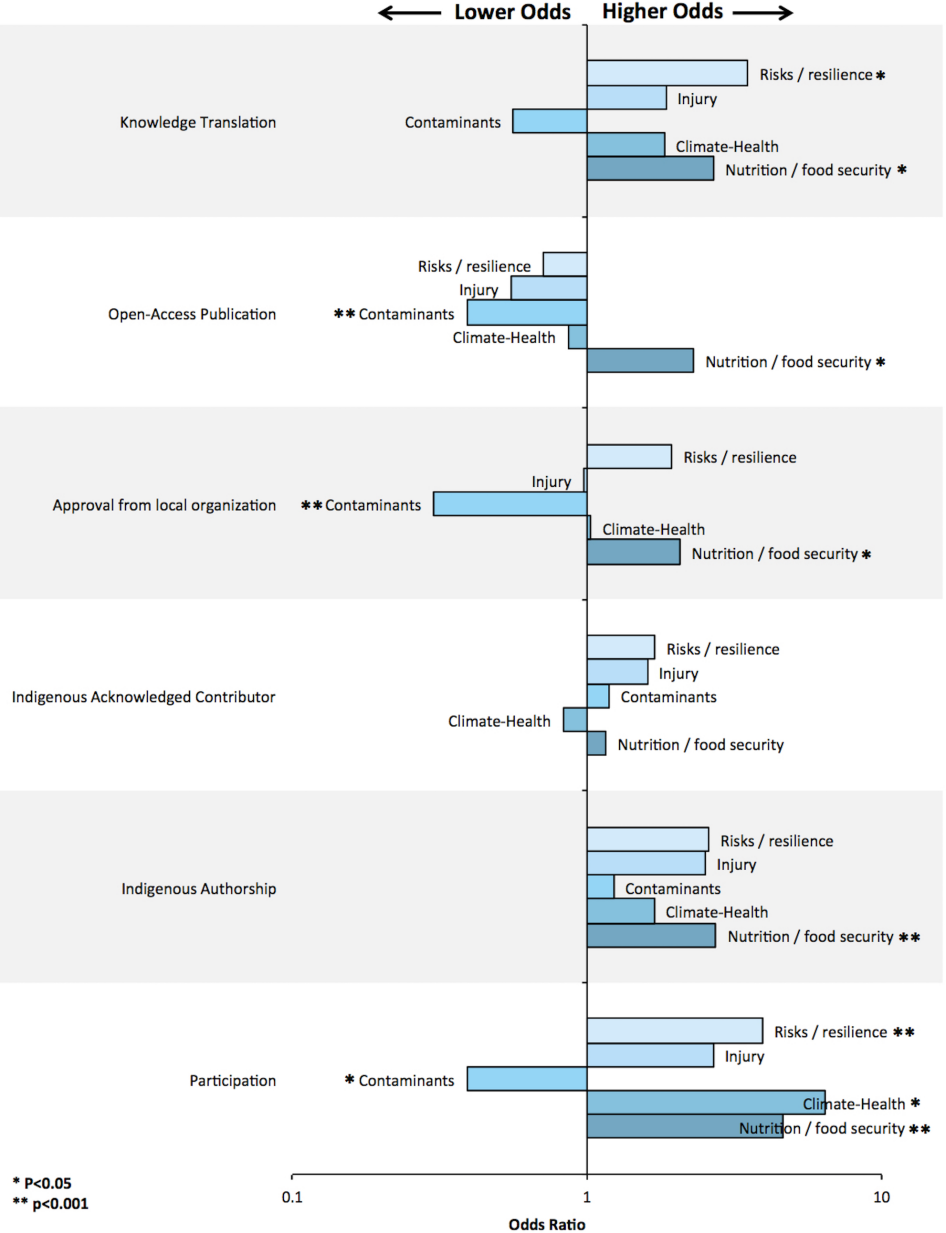
Multivariable exact logistic regression examining associations between research topic and various indicators of community-engaged research.

**Fig 7:**
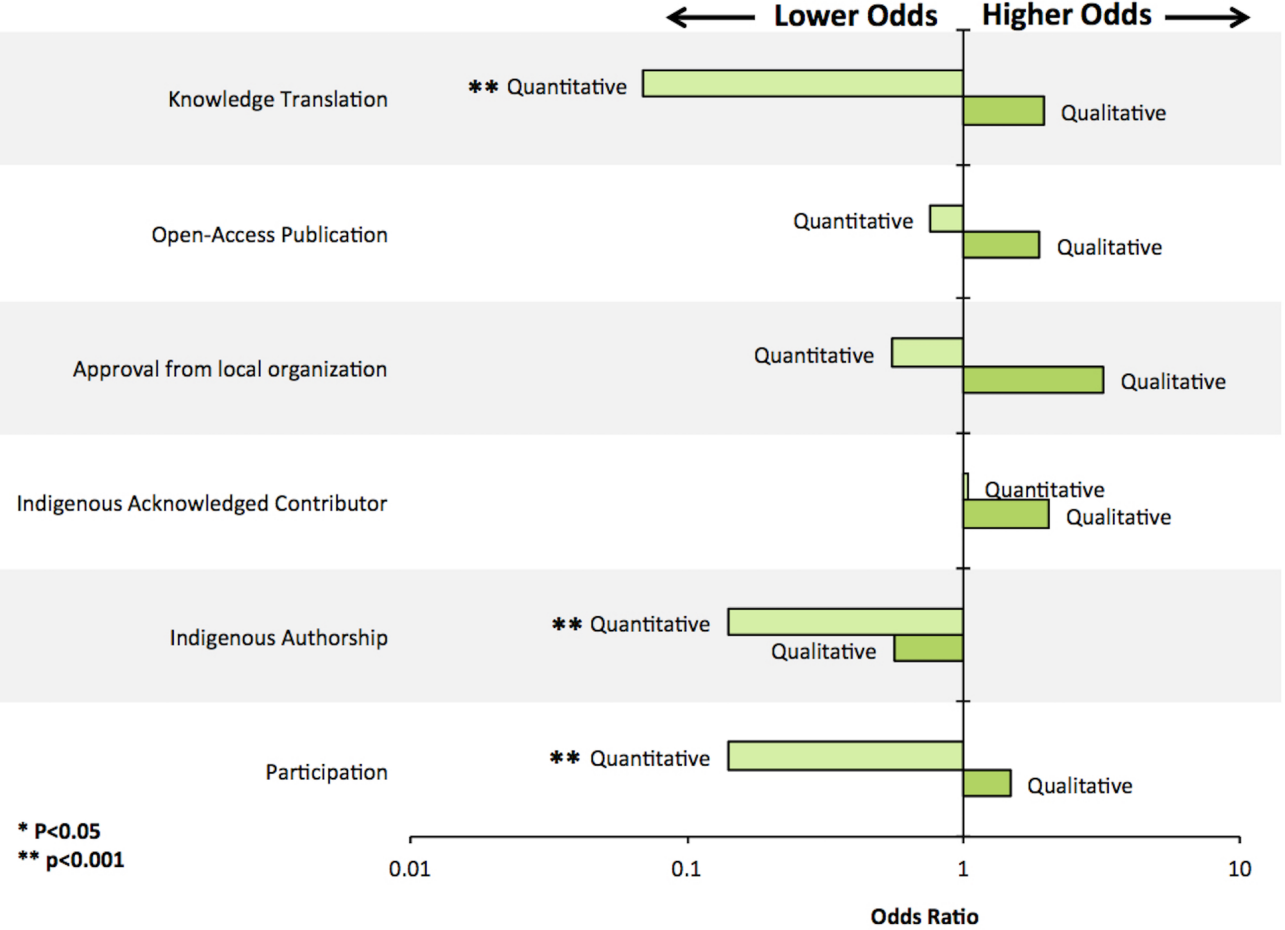
Multivariable exact logistic regression examining associations between research method and various indicators of community-engaged research.

Of all the environment-related Indigenous health research articles (n = 210 articles), some publications mentioned using participatory, community-based research (CBPR) methods to engage the community in the study (38.1%) (Table 3). A modest, but statistically significant, increase in articles reporting CBPR methods was observed over time (Fig 3). Research conducted in Nunatsiavut was significantly more likely to report CBPR methods (p<0.05, Fig 4). While Nunavik had one of the highest number of environment-related Indigenous health research articles published, it was significantly less likely to report using CBPR methods (9/58 articles; 16%; p<0.05; Figs 4 and 5). Articles about nutrition and food security, climate change impacts on health, and environmental risks and resilience had significantly higher odds of reporting CBPR methods when compared to other topics (Figs 4 and 6; p<0.05). For example, while research about environmental contaminants was the most common research topic, it was significantly less likely to report CBPR methods compared to other topics (Figs 4 and 6; p<0.05). While quantitative methods were used in the large majority of health and environment articles (158/210 articles; 75%; Fig 4), they were significantly less likely to report using CPBR methods compared to qualitative research (40/158 articles; 25%; Fig 7; p<0.05).

Of the articles identifying the engagement of the community in the research (n = 39), information detailing how the regional or local Indigenous governing organization was involved, oversaw, or made aware of the research beyond citizens being research subjects was not consistently noted. If noted, this information was in the background to the research (n = 16/39) [44–58] the method section (n = 29/39) [47, 48, 50–52, 54, 55, 57, 59–79], and/or in the results section (n = 3/39) [59, 74, 80] of the articles. A number of diverse ways were employed to identify the role of the local or regional Indigenous governing organization in the research including the development of a research or advisory board [55, 65, 75, 77, 81]; identifying research as a ‘collaboration’ or ‘partnership’ with an Indigenous organization or government [44, 46, 48, 50, 51, 55, 56, 61, 62, 66, 73, 76, 79, 82]; and/or noting a workshop was hosted to engage or share information with the Indigenous government or organization [52, 62, 63]. Of the articles that detailed the role of community in the research, 33% (n = 13/39 articles) explicitly indicated that the research was community-initiated or community-led [46, 48, 51, 53, 55, 57, 60, 65, 67, 71, 80, 81, 83]. Community-initiated or -led was described as awarding the research funding to a local Indigenous government [53, 60], the principal investigator being the local Indigenous government [46], or a collaboration resulting from a previous working relationship between the academic researchers and the community [46, 51, 72].

#### Indigenous authorship was uncommon

Indigenous authorship on papers increased over time, with Nunatsiavut, Nunavut, Yukon, and Alaska having higher proportions of articles authored by Indigenous people, organizations, or governments; however, these differences were not statistically significant (p<0.05). Research reporting on nutrition and food security were significantly more likely to have Indigenous authorship compared to other topics (Fig 6; p<0.05), while research reporting the use of quantitative methods was significantly less likely to have Indigenous authorship compared to qualitative research (Fig 7; p<0.05).

Of the articles detailing the engagement of a community within the research (n = 39 articles), 74% (n = 29) listed a co-author with affiliation to an Indigenous community, government, or organization. Examples of author affiliation included: Indigenous governments [46, 47, 51–53, 55–57, 60, 65, 71–73, 75, 80, 83]; Indigenous focused health boards [48, 50, 58, 59, 64, 70]; community liaisons [44, 66, 76]; or an Indigenous-led research organization [51, 67, 68, 79]. In 10% (n = 4) of these articles, the name of the Indigenous organization that partnered in the research (and not an individual representing the Indigenous organization), was listed as the author [53, 56, 71, 83]. 77% (n = 30) of articles acknowledged the regional or local Indigenous government or organization in an acknowledgment section [47, 48, 51–53, 55, 56, 58, 60–66, 68–71, 73–76, 78–84]. Only one article (1/39 articles) did not include an Indigenous author affiliation or an Indigenous acknowledged contributor [49].

#### Community consent was inconsistently identified

Over time, there were modest but significant increases in reporting that Indigenous organizations granted permission to conduct research (Fig 3; p<0.05). Research conducted in Nunatsiavut was significantly more likely to report the granting of local permission to conduct research compared to other regions (Fig 5; p<0.05). While research on nutrition and food security were significantly more likely to report granting of local permission to conduct research, research on environmental contaminants was significantly less likely to report this permission compared to other research topics (Fig 6; p<0.05).

Of the articles that provided detailed information about engagement of a community in the research process, 56% (n = 22/39) explicitly described receiving research approval or permission from an Indigenous community, government or organization [44, 50, 51, 55, 57, 59–61, 63, 65–67, 69, 71–74, 76, 77, 80, 82, 83]. Additionally, 18% (n = 7/39 articles) did not clearly articulate the process of gaining permission by a local organization, but instead inferred community permission [46, 48, 53, 56, 68, 79, 81]. The descriptions of inferred permission varied and included: collaborating with an Indigenous government or community and/or working with a local advisory board [44, 46, 48, 50, 55, 56, 61, 65, 66, 71, 73, 75–77, 79, 81, 82]; a joint research-related intervention with an Indigenous organization [46, 53, 60, 65, 75, 80, 83]; a signed agreement with the community [56, 68, 77]; and/or articulating the role of the Indigenous government or organization in the title of article [46]. The descriptions of local permission were primarily reported in the article’s methods section, but also in an ethics section [63], a protocol section [78], and the background section [55, 79].

### Were research results used by Indigenous communities, governments, or organizations?

Few articles reported on sharing the results with the community (n = 31/210 articles; 14.8%; Table 3); however, modest, but not significant, increases were observed over time (Fig 3; p>0.05). Research conducted in Nunavik, Nunavut, and Alaska were significantly less likely to report on knowledge mobilizations compared to other regions (Fig 5; p<0.05). Research on nutrition and food security as well as environmental risk and resilience were significantly more likely to report how results were shared compared to other research topics (Fig 6; p<0.05). Research using quantitative methods were significantly less likely to report how the research results were shared compared to qualitative research (Fig 7; p<0.05).

Of the articles that detailed the engagement of a community in the research, many describe the dissemination of results (66.7%, n = 26/39) [44, 46, 48, 50–53, 55–57, 60–62, 65–67, 71, 73, 75–77, 79–83]; however, there was no consistent method or section of the paper that this information commonly appeared. Examples of research mobilization include locally published booklets, reports, or DVDs [48, 50, 53, 75, 80]; and presentations to an Indigenous government [76].

### Can Indigenous communities, governments, and organizations access research results?

A modest decrease in the number of open-access articles was observed over time (Fig 3; p>0.05). Nutrition and food security research was significantly more likely to be open-access; and research about environmental contaminants was significantly less likely to publish open-access articles compared to other research topics (Fig 6; p<0.05).

Of the articles that detailed community-engaged methods, the majority of articles (87%, n = 34/39) were accessible through some form of open-access [44, 46, 48–53, 55–60, 62, 63, 65–72, 75–84]. Of these open-access articles, 23% (n = 9/34) were published in the *International Journal of Circumpolar Health* [46, 48, 52, 62, 63, 68, 69, 76, 77]; 8% (n = 3/34) in *EcoHealth [50, 53, 56];* 8% (n = 3/34) in *Social Science and Medicine* [55, 60, 71]; and 5% (n = 2/39) in *Arctic* [49, 75]. The remaining 50% (n = 17/34) of open-access articles were either published in another open-access journal or a journal that has an open-access option (n = 7/34) [59, 60, 65, 67, 70, 80, 81], or made publically available through a third party host (n = 10/34) [44, 50, 51, 57, 58, 66, 78, 79, 82, 84].

## Discussion

Our findings suggest that reporting the relationship between researchers and Indigenous partners is not common practice in peer-reviewed environment-related health research in the Circumpolar North and, therefore, is not fully responding to a changing social and political climate in the North, which calls for the recognition of knowledge creation as a form of self-determination [17, 21]. This is an important consideration in challenging the legacies of on-going colonization. The creation of research as a form of self-determination is evident in the creation of self-governed territories, land claim settlement areas, and Indigenous health authorities, and has challenged the way in which decisions affecting the health of Indigenous peoples are identified and addressed [16, 85, 86]. As Indigenous communities are positioned to determine what research is required to support and promote the health of their populations, the role of the researcher and the research they produce is also changing. As reported in a number of articles in this SRLR, Indigenous governments and organizations are in the position to seek out the expertise to support their own research agendas [46, 48, 51, 53, 57, 60, 61, 67, 80, 81]; yet, few of these articles had Indigenous authorship.

While research may be responding to community-defined priorities, we found this is not frequently reported in peer-reviewed journal articles. In Canada, reporting on Indigenous leadership in defining the research is important given the recent national discourse on reconciliation and calls from Indigenous peoples to actively decolonize research and ensure that Indigenous needs, priorities, and ways of knowing are respected, responded to, and included [21, 87, 88]. A key question from this research then becomes: how do non-Indigenous researchers demonstrate their awareness of the changing socio-political environment and recognize the role of research in decolonization and reconciliation processes, and reflect and communicate this through peer-reviewed journal articles?

Of note, articles reporting CBPR methods increased over time. Tenets of CBPR are generally understood to align with Indigenous research ethics, which include developing a relationship with the community, attending to a process of mutual learning, and conducting research that responds to priorities identified by communities [21, 26, 32, 89]. However, the application and interpretation of such tenets vary among those using CBPR methodologies [17, 89]. We attempted to capture these variations, and analysed the degree of detail provided in the articles. For example, in one article, the authors outlined the engagement process and clearly indicated that the research was directed, overseen, and was important for the community [46]. A different approach in another article involved the authors stating that the research reflected the interests of an Indigenous government and was the outcome of an ongoing relationship [73]. These are two different approaches to identifying the role of the Indigenous governments in directing the research; however, both methods were published in a peer-review journals, identified the use of participatory or community engagement methods, and inferred knowledge, consent, and participation of the community. The reporting of this information helps clarify why the research was conducted, its relationship to the Indigenous community, and relevance for the community.

Quantitative research was less likely to report any metric of community engagement that we examined in this review. These findings suggest that quantitative research journals do not require, encourage, and/or reward reporting on these methodologies, and reflects quantitative health research not developing, or having been slow to develop, culturally safe guidelines or methods for active community participation [20]. It could further suggest that those working within this methodology are not fully considering or understanding knowledge creation as a process of reinforcing a colonial construct of health researchers as knowledge creators, and Indigenous peoples as subjects [21, 88, 90]. This is particularly germane for all health research, which is historically grounded in positivism and often considered neutral and value free, denying recognition that decisions made are imbued with underlying assumptions, which historically, are based in Western epistemologies and approaches [21, 33].

Given these realities, can it be expected that reporting community-engaged indicators could readdress the role of research in colonizing Indigenous peoples? It might be suggested that this is a contributing factor to actively work towards decolonizing health research. Indeed, outlining the role that Indigenous organizations played in the development of research shifts the gaze to where power is generated and held, and challenges the generation of knowledge as solely the domain of academic institutions [20, 21]. Further, Indigenous governments and organizations can benefit from research that utilizes quantitative and/or qualitative approaches. If this is generally understood and accepted, this research supports the position that a move towards detailed reporting of the role of Indigenous organizations and researchers should occur, regardless of research approach.

Also illuminated in our review is the lack of systematic or unifying method used to communicate how health research is responding to community-identified issues, and how the research has been used or applied by Indigenous communities. This absence could reflect the reality that describing and interpreting if and how a research project is indeed responding to community-identified issues is both difficult to achieve and discern. Furthermore, this can also be challenging given journal word count limits. Articles in this review, specifically those applying tenets of CBPR, provide insight into how researchers can better communicate if and how health research is responding to Indigenous-identified priorities. A number of indicators that communicated the relationship between the research project and the Indigenous government or organization were identified in this SRLR. Depending on the requirements of the journal, this information could be placed in the introduction, methods, or ethics approval section. In the discussion or conclusion of an article, alongside suggestions for future research ideas, acknowledgment of the way in which results were mobilized with the partnering Indigenous organizations may also communicate the relationship between research project and Indigenous communities.

Article authorship rarely included Indigenous authors [53, 56, 71, 83]. The practice of identifying the community, government, or organization as an author, as opposed to an individual representing a community, government or organization, avoids essentializing or silencing Indigenous participation in research. The practice, if acceptable to the community, government, or organization, is a challenge to recognizing authorship as defined by academic institutions. Consideration of the limitations of ‘recognition’ [87, 91, 92], then, may well provide the field of Circumpolar Indigenous health research further insight into who is defining Indigenous identity and how this shapes, upholds, challenges, or dismantles the distribution of power. Furthermore identifying a community, government, or organization as the author–while fraught with its own dilemmas about community and what constitutes community [89, 92],–ensures that the community is acknowledged as a contributor to the research.

This SRLR also explored how Indigenous governments or organizations are able to access research that has been conducted in the communities they serve, vis-à-vis some form of open-access. While the number of open-access articles was the only metric that decreased over time, what is not told through this metric is whether Northern Indigenous organizations are, indeed, accessing and using the peer-reviewed articles. We acknowledge that the ‘open access’ status of an article does not serve as an effective indicator of how the research was used by or served the involved community. This is not to suggest that current requirements and guidelines published by funding agencies requiring articles to be available within 12 months of publishing through open-access [93] is not a welcome move. Further research into this open-access query may illuminate the role of peer-review articles in informing Northern Indigenous directed policies and programs.

Finally, it is important to note that this literature review only assessed what authors wrote in their articles. We recognize that the absence of reporting community-engaged methods does not presume that the research did not genuinely engage with an Indigenous organization, respond to a community priority, work with an Indigenous organization to develop and/or implement the research project being reported. The absence of reporting community-engaged methods, for instance, could reflect journal word count limits, criteria for authorship, and other requirements imposed by academic journals. Indeed, this may bias what information gets published in peer-reviewed or grey literature.

## Conclusion

We sought to determine if and how environment-related Indigenous health research conducted in the Circumpolar North communicates its role in serving communities in peer-reviewed journal articles. The small number of articles detailing community-engaged methods and the lack of unifying methods used to describe the relationship between the research project and the health research priorities of Indigenous governments or organizations give indication that reported health research is not keeping pace with a changing North. Notably, the predominance of qualitative research articles reporting community-engaged metrics could suggest that the importance of applying and reporting the tenets of CBPR is better understood among qualitative researchers than quantitative researchers. This could also suggest that CBPR approaches are better developed for qualitative research, and that methods that engage with the community and respond to community priorities needs to be further developed for quantitative health research.

Processes and impacts from the legacies of colonization endure to this day, and the demand for ethical health research that responds to community-defined and community-identified priorities is part of other growing movements across North America and, indeed, worldwide. The *Idle No More* movement, the hunger strike by Chief Theresa Spence of the Attawapiskat First Nation, the #MakeMuskratRight campaign, and the Truth and Reconciliation Commission Reports and related Calls to Action [94] are all movements by Indigenous leaders, communities, governments, and organizations that call for change in the relationship with Indigenous peoples. This call extends to those working in the area of health research; yet, how is this demonstrated to peers and those consuming peer review research?

Our findings concur with calls made for greater methodological transparency in peer reviewed literature involving Indigenous research participants or interests [17, 29]. To this end, we offer a number of indicators that should be reported in peer-reviewed articles to begin conveying to the reader the author’s knowledge of conducting ethical research with Indigenous communities and the manner in which the Indigenous community was engaged:

1. Recognize the Indigenous government or organization as an author;
2. Identify whether community permission to conduct the research was granted;
3. Describe the relationship of the Indigenous government or organization to the research project (i.e., initiator, principal investigator, collaborator);
4. Acknowledge the contributions of the advisory board; and/or
5. Identify and describe the role of the community in the article and abstract, and not solely in the acknowledgments section.

These indicators are not provided as a prescriptive or final check-list; rather, they serve to inspire a broader discourse on the issues of conducting health research with Indigenous populations. Considerations of how research is reported can contribute to de-essentializing Indigenous communities and raise awareness of how on-going colonization mediates Indigenous health status. Just as the methods used to address complex issues are diverse, so too are the people, findings, and application of the findings. Furthermore, there is no one way of approaching the health issues of the North, nor is it assumed there is one way of reporting on research conducted in the North. Engaging in an anti-colonial discourse, however, requires consideration of the role of reporting and what is reported in peer review journals. This requires the active participation of a number of players including health researchers of all methodologies, their students, the funders, the communities, and editorial boards of peer review journals.

## Supporting information

S1 Fig Flowchart for qualitative analysis. (TIFF)

S1 Table Data extraction questions. (DOCX)

S2 Table Prisma checklist. (DOCX)
