# Supplementary figures and images for: Who is research serving? A systematic realist review of circumpolar environment-related Indigenous health literature
Source: PLoS One. 2018 May 24;13(5):e0196090. doi: 10.1371/journal.pone.0196090 (PMC5993119; doi:10.1371/journal.pone.0196090)

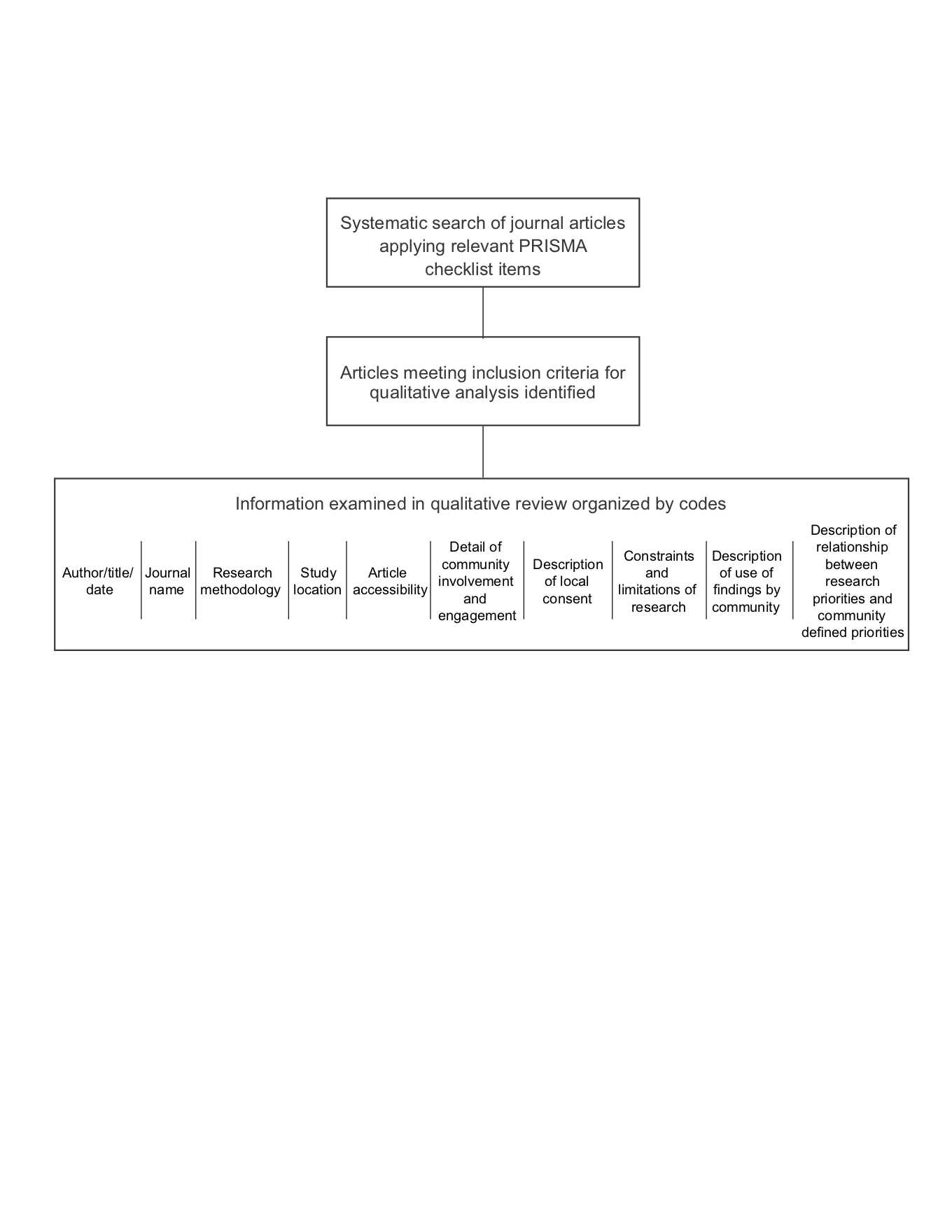

Supplement: S1 Fig — (TIFF) [file pone.0196090.s001.tiff]
