# Supplementary material for: Who is research serving? A systematic realist review of circumpolar environment-related Indigenous health literature
Source: PLoS One. 2018 May 24;13(5):e0196090. doi: 10.1371/journal.pone.0196090 (PMC5993119; doi:10.1371/journal.pone.0196090)
Supplement: S1 Table — (DOCX) [file pone.0196090.s002.docx]

**What region was this research conducted in (select all that apply)?**

- Nunatsiavut (Nain, Hopedale, Makovik, Postville or Rigolet)
- Nunavut
- Northwest Territories
- Nunavik
- Yukon
- Alaska
- Other

**What year was the article published?**

- 2000
- 2001
- 2002
- 2003
- 2004
- 2005
- 2006
- 2007
- 2008
- 2009
- 2010
- 2011
- 2012
- 2013
- 2014
- 2015
- Other

**What year was the data collected?**

- 2000
- 2001
- 2002
- 2003
- 2004
- 2005
- 2006
- 2007
- 2008
- 2009
- 2010
- 2011
- 2012
- 2013
- 2014
- 2015
- Other

**What was the topic of the health research (select all that apply)?**

- Nutrition and food security
- Climate change impacts on health
- Environmental contaminants or monitoring contamination
- Intentional and unintentional injury
- Environmental risks and resilience
- Other

**What study methods were used?**

- Qualitative methods only (e.g. interviews, focus groups, photovoice, qualitative analysis (thematic analysis, grounded theory, data are typically words, *and so on*)
- Quantitative methods only (e.g. surveys, biological samples, statistical analyses, descriptive statistics, data are numbers, *and so on*)
- Mixed qualitative and quantitative methods (e.g. a mix of both methods)
- Other

**Did the authors state that they used participatory, community-based, or other methods that engage the community in the study design, data collection, or data analysis?**

- Yes
- No
- Unsure

**Was the article open-access/freely available?**

- Yes
- No
- Unsure

**Was an Indigenous organization listed as a co-author?**

- Yes
- No
- Unsure

Not included as an Indigenous affiliation were Yukon Government, Government of the Northwest Territories and Alaska Public Health. While both organizations work to better the health of Northern Indigenous populations they do not identify as regional Indigenous organizations but rather organization working to support all Northerners, Indigenous and non-Indigenous.

**Did the article articles explicitly describe seeking and/or receiving research approval or permission from a regional or local Indigenous government or organization responsible for the research in the region?**

- Yes
- No
- Unsure

**Did the article clearly report if/how the results were shared with the community?**

- Yes
- No
- Unsure

**Did the article acknowledge any community members or governments in the acknowledgment section?**

- Yes
- No
- Unsure
